# Downregulation of developmentally regulated endothelial cell locus-1 inhibits the growth of colon cancer

**DOI:** 10.1186/1423-0127-16-33

**Published:** 2008-12-25

**Authors:** Xiaolong Zou, Haiquan Qiao, Xian Jiang, Xuesong Dong, Hongchi Jiang, Xueying Sun

**Affiliations:** 1 The Hepatosplenic Surgery Center, Department of General Surgery, The First Clinical College of Harbin Medical University, Harbin 150001, PR China; 2 Department of Molecular Medicine & Pathology, Faculty of Medical and Health Sciences, University of Auckland, Auckland, New Zealand

## Abstract

Developmentally regulated endothelial cell locus-1 (Del1) is an embryonic angiogenic factor expressed in early embryonic endothelial cells, but recently has been found to be expressed in some forms of cancers including colon and breast cancers, and melanoma, and human cancer cell lines. Overexpression of Del1 accelerates tumor growth by enhancing vascular formation, implying Del1 may be a potential target for anti-angiogenic cancer therapy. The study aims to investigate whether downregulation of Del1 could inhibit the growth of tumors established in nude Balb/c mice by subcutaneous implantation of human LS-174T colon cancer cells. The shRNA expression vectors targeting human Del1, and vascular endothelial growth factor (VEGF) were constructed. Gene transfection of Del1-shRNA downregulated expression of Del1 in LS-174T cells *in vivo *and *in vitro*, but did not alter the proliferative or survival properties of cells *in vitro*. Gene transfection of VEGF-shRNA downregulated expression of both VEGF and Del1 in LS-174T cells *in vivo *and *in vitro*. Both Del1-shRNA and VEGF-shRNA gene therapies exhibited anti-tumor activities and they also showed a synergistic effect in suppressing growth of colon tumors by anti-angiogenesis and anti-proliferation. Although further investigation to clarify the mechanisms explaining the role of Del1 in tumor growth, and the interaction between VEGF and Del1, is required, the results indicate that downregulation of Del1 presents a potent therapeutic strategy to combat colon cancer.

## Introduction

Colon cancer is the fifth cause of cancer-related death in developed countries, and its incidence is rising at an alarming rate in developing countries [1]. Unfortunately, the conventional adjuvant treatments have shown only modest effects on long-term survival after surgical resection. There is, therefore, an urgent need to seek novel therapies to treat colon cancer. Like all the other solid neoplasms, colon cancer depends on the process of angiogenesis, the formation of blood vessels, for both local and metastatic growth beyond a few cubic millimeters, which provides the rationale for antiangiogenic therapy aimed at targeting the tumor blood supply [2]. Inhibition of angiogenesis has become an attractive target for cancer therapy because it theoretically offers the hope of long-term control of neoplasm progression [3].

Tumor angiogenesis is a multi-step process, in which the switch to the angiogenic phenotype requires both upregulation of angiogenic stimulators and downregulation of angiogenic inhibitors [3]. Developmentally regulated endothelial cell locus 1 (Del1) has recently been identified as a new angiogenic factor [4]. The Del1 protein encoded in this locus contains three epidermal growth factor (EGF)-like repeats and the second EGF repeat contains an RGD motif, and through interaction with integrin αvβ3, Del1 mediates endothelial cell attachment and migration. Attachment to Del1 leads to clustering of integrin receptors, focal contact formation, and the phosphorylation of signaling molecules such as p125FAK and MAP kinase [5]. In a chick chorioallantoic membrane assay, Del1 was found to be a potent angiogenic factor, and its angiogenic activity requires αvβ3 receptor activation [5].

A striking feature of Del1 expression is that it begins to decline after the endothelial cell contributes to vascular formation and disappears completely by birth [4]. Del1 expression is identified in tissues of brain, heart, small intestine and kidney, but not colon, liver, or lung, in human adult [6]. However, Del1 has been found to be expressed in some forms of primary human cancers including breast cancer, colon cancer and melanomas, even the original tissues do not express Del1[7], and in many tumor cell lines [6,8]. Overexpression of Del1 after gene transfection accelerated tumor growth by reducing apoptosis of tumor cells and increased tumor vascularization [7]. The data imply Del1 could be a potential target for cancer anti-angiogenic therapy. It has also demonstrated that Del1 was induced by tumor-derived vascular endothelial growth factor (VEGF), and anti-VEGF antibody inhibited this induction [9]. Therefore, we designed this study to investigate whether downregulation of Del1 with shRNA targeting Del1 and VEGF could inhibit tumor growth in a mouse model of human LS-174T colon cancer.

## Materials and methods

### Mice, cell lines and antibodies

Male 6-week-old Balb/c nude mice (nu/nu) were purchased from the Animal Research Center, The First Clinical Medical School of Harbin Medical University, Harbin, China. The human colon cancer LS-174T and HT29 cells were kindly presented by Professor Daling Zhu, the Pharmacy College of Harbin Medical University. Cells were grown in RPMI 1640 medium containing 10% fetal calf serum (FCS) in 5% CO_2 _humidified atmosphere at 37°C. The antibodies used in this study included anti-Del1 Ab (Novus Biologicals Inc, Littleton, USA), anti-VEGF and anti-Ki-67 Abs (Santa Cruz Biotechnology, Inc, CA, USA), and anti-CD31 Ab (Pharmingen, CA, USA).

### Construction of shRNA expression plasmids

The Del1-shRNA (short hairpin RNA) and VEGF-shRNA expression plasmids and the negative control plasmid were constructed by Genesil Biotechnology Co, Ltd (Wuhan, China). Four different sites for small interfering RNA (siRNA) targeting human Del1 gene (Genbank HSU70312): AATGGAGGTATCTGTTTGCCA (207–227 nt), GTTCTAGTGTTGTGGAGGT (286–304 nt), AAGCATACCGAGGGGATACAT (388–408 nt), and AATGTCATCGACCCTCCCATC (1443–1463 nt), and three sites for siRNA targeting human VEGF gene (GenBank NM_001033756): AGAAAGATAGAGCAAGACA (1429–1447 nt), CGCGAGAAGTGCTAGCTCG (728–746 nt) and CCTTGCCTTGCTGCTCTAC (1064–1082 nt), were designed, respectively. BLAST was performed to ensure that the siRNAs did not have significant sequence homology with other genes. The synthesized oligonucleotides were inserted into the expression vector, pGenesil-4, which contains four promoters including mU6, hU6, hH1, h7SK, and an eGFP fragment. An expression vector containing the non-specific siRNA was designed as a negative control, of which the sequence was 5'-GACTTCATAAGGCGCATGC-3'. The expression plasmids have been confirmed the integrity of the expression plasmids was confirmed by DNA sequence analysis.

### Animal experiments

All the procedures had been approved by the institutional Animal Care and Use Committee of Harbin Medical University. LS-174T cells were cultured, grown to 60–70% confluence and harvested. 5×10^6 ^cells in 0.1 ml PBS were s.c injected into the left flank of the mice. Tumor size was measured with a caliper, and tumor volumes were estimated according to the formula: *π*/6 × *a*^2 ^× *b*, where *a *is the short axis, and *b *the long axis. When the size of tumors reached to ~100 mm^3 ^in volume, the mice were randomly assigned to treatment groups (each group had 12 mice): Control, Del1-shRNA, VEGF-shRNA and Del1-shRNA + VEGF-shRNA. The mice received intratumoral injection of 200 μg of plasmids diluted in 100 μL of FuGENE™ 6, which was shown to be an efficient *in vivo *transfection reagent in our previous study [10]. For combinational treatment, reagents were delivered in a timed fashion, where Del1-shRNA plasmid was injected first, followed by VEGF-shRNA plasmid 12 h later. Four mice from each group were sacrificed 4 days after gene injection and tumors excised for detecting gene expression. The remaining mice were monitored for 3 weeks after gene injection and culled, tumor excised for further analysis.

### Immunohistochemical analysis

The formalin-fixed tumor tissues were embedded and sectioned (5μm). Slides were deparaffinized in xylene and rehydrated with graded ethanol. The sections were digested with proteinase K for 15 min, followed by blocking with 3% H_2_O_2 _in methanol for 10 min. After washing, the sections were further blocked with serum for 2 h, and incubated overnight with primary Abs. They were subsequently incubated for 30 min with appropriate secondary Abs using the Ultra Sensitive TMS-P kit (Zhongshan Co., Beijing, China), and immunoreactivity developed with Sigma FAST DAB (3,3'-diaminobenzidine tetrahydrochloride) and CoCl_2 _enhancer tablets (Sigma-Aldrich, Shanghai, China). Sections were counterstained with hematoxylin, mounted, and examined by microscopy.

### Assessment of tumor vascularity

The method has been described previously [11]. Briefly, tumor sections were immunostained with an anti-CD31 Ab as described above. Stained vessels were counted in five blindly chosen random fields at 400 × magnification under microscopy, and the mean microvessel density was recorded.

### Quantitation of Ki-67 proliferation index

The tumor sections were immunostained with anti-Ki-67 Ab as above, and the Ki-67 positive cells were counted in 10 randomly selected × 400 high-power fields under microscopy. The Ki-67 proliferation index was calculated as the following formula: the number of Ki-67 positive cells/the total cell count × 100%.

### Western blot analysis

The method has been described previously [11]. Briefly, tissues were homogenized in protein lysate buffer. Debris was removed by centrifugation. Protein samples (30 μg) were resolved on 12% polyacrylamide SDS gels, and the proteins electrophoretically transferred to polyvinylidene difluoride (PVDF) membranes. The membranes were blocked with 3% BSA overnight, incubated with primary Abs, and then with an alkaline phosphatase-conjugated secondary Ab, and immunoreactivity visualized with 5-bromo-4-chloro-3-indolyl phosphate (BCIP)/nitro blue tetrazolium (NBT) (Tiangen Biotech Co. Ltd., Beijing, China). Blots were stained with an anti-β-actin Ab to confirm that each lane contained similar amounts of tumor homogenate. The density of each band was evaluated using the gel image analyzer. The relative density of was calculated as the following formula: band density/β-actin band density.

### *In vitro *transfection and Western blot analysis

Cells were seeded in RPMI 1640 medium supplemented with 10% FCS in 10 cm plastic dishes. Upon reaching 60–70% confluence, they were transfected with 4 μg of plasmid using lipofectamine PLUS™ (Life Technologies, China). Forty-eight hours later, cells were detached with trypsin, lysed on ice with lysis buffer (50 mM Tris-Cl, pH 7.5, containing 1% v/v Triton-X, 15% v/v deoxycholic acid, 0.5 M NaCl, and 1 mM EDTA), and subjected to Western blot analysis as above.

### RNA isolation and RT-PCR analysis

Total RNA was extracted from the transfected cells above using TRIZOL according to manufacturer's protocol. For first-strand cDNA synthesis, 1 μg of total RNA was incubated at 42°C for 50 min in 10 μl reverse transcription buffer containing 20 U of Rnasin, 0.05 μg of Oligo (dT), and 50 U super Script TM II RT. The reaction was stopped by heating the mixture at 94°C for 5 min. A 354 bp PCR product was amplified from Del1 major isomer gene with a pair of primers: 5'-CGTCTGGCTCTTGGTCGG-3' and 3'-TTCTTTAACAGTTATGTTTACG-5'; a 208 bp PCR product was amplified from VEGF_165 _with a pair of primers: 5'-TTGCCATTCCCCACTTGA-3' and 3'-ACTCACTGGACGAAAACC-5'; and a 497 bp PCR product was also amplified from GAPDH cDNA as an internal control, with a pair of primers: 5'-GAAGGTGAAGGTCGGAGT-3' and 3'-TGAAACCATAGCACCTTCC-5'. The reaction mixture was first heated at 93°C for 4 min and the amplification was carried out for 30 cycles at 93°C for 30 s, 58°C for 30 s, and 72°C for 30 s, followed by incubation at 72°C for 5 min. PCR products underwent a 1.5% agarose gel electrophoresis, and the density of each band was evaluated using the gel image analyzer. The relative density of mRNA levels was calculated as the following formula: band density/GAPDH band density.

### Cell viability and serum starvation assay

The transfected cells from above were counted and seeded into a 96-well plate (3×10^3^/well) in 100 μl of RPMI 1640 with 10% FCS and incubated for the time periods indicated. 20 μl of the MTT reagent was added, cells were further incubated at 37°C for 1 h, and then the OD of supernatants was determined in a microplate reader at 490 nm. Each experiment was performed in triplicate and three individual experiments were performed. For serum starvation apoptosis assay, transfected cells were seeded into a 96-well plate (6×10^3^/well) in 100 μl of RPMI 1640 with 10% FCS and incubated overnight to allow attachment. The following day, medium was replaced with serum-free RPMI 1640 medium and cells were incubated for various time periods. The cell viability was measured by MTT assay as described above.

### Statistical analysis

Results were expressed as mean values ± standard deviation, and Student's t-test was used for evaluating statistical significance. A value of P < 0.05 was considered significant.

## Results

### Expression of Del1 in colon cancer cell lines and tissues from patients

**Figure 1:**
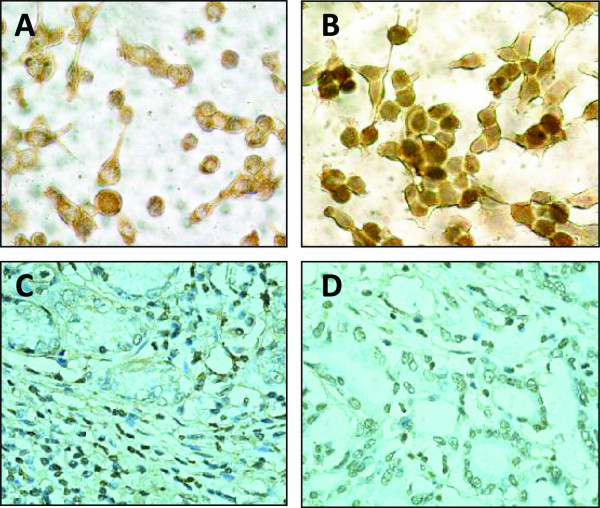
**Immunohistochemical analysis of colon cancer cells and tissues**. Illustrated are representative photographs of LS-174T (A) and HT29 (B) cells, and two colon tumor sections from two individuals (C and D), immunostained brown with an anti-Dle1 Ab.

Firstly, we examined the expression of Del1 protein in human colon cancer LS-174T and HT29 cells by immunohistochemical analysis as shown in Figure 1A (LS-174 cells) and 1B (HT29 cells). We also immunostained the colon cancer tissues from 10 patients with anti-Del1 Ab and Del1 was found to be expressed in all the colon cancer tissues (10/10) at various levels. Two representative photographs of tumor sections from two colon cancer patients were shown in Figure 1C and 1D.

### Downregulation of gene expression by shRNA gene transfection *in vivo*

**Figure 2:**
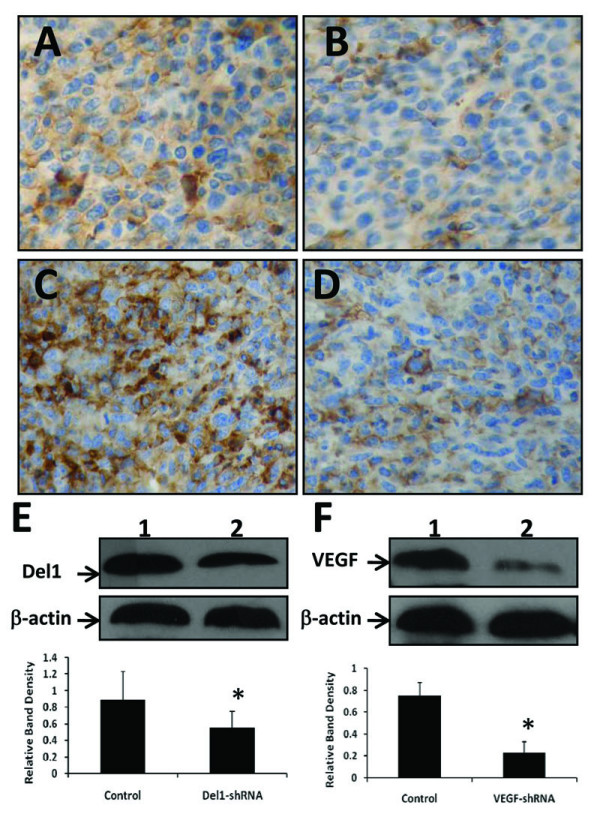
**shRNA gene transfection downregulates gene expression *in vivo***. Established LS-174T tumors (~100 mm^3 ^in volume) were injected with either control vector (A, C), Del1-shRNA (B) or VEGF-shRNA (D) expression plasmids. Illustrated are representative tumor sections prepared 4 days following gene transfer, stained brown with Abs against Del1 (A, B) or VEGF (C, D). Tumoral expression of Del1 (E) or VEGF (F) was detected by Western blot analysis. Blots of homogenates from tumors injected with control vector (lane 1) and shRNA plasmid (lane 2) were stained with Abs against Del1 (upper row) (E), or VEGF (upper row) (F), and an anti-β-actin Ab (lower row). The density of each band was measured and normalized to that of β-actin. *Indicates a significant difference at *P *< 0.05 in the band intensities of Del1 or VEGF between control vector transfected groups.

A DNA/FuGENE™ 6 transfection vehicle containing shRNA expression vectors was injected into established LS-174T tumors (~100 mm^3^). Immunohistochemical staining of tumor sections prepared from tumors 4 days after transfection revealed that Del1 was intensely expressed in control vector-treated tumors (Figure 2A), whereas Del1-shRNA transfection resulted in markedly downregulation of Del1 *in situ *(Figure 2B). The results were confirmed by Western blot analysis (Figure 2E). Similarly, gene transfection of VEGF-shRNA greatly downregulated expression of VEGF *in situ *(Figure 2C vs. Figure 2D) as revealed by immunohistochemical analysis and confirmed by Western blot analysis (Figure 2F).

### Del1-shRNA and VEGF-shRNA synergizes to suppress tumor growth

**Figure 3:**
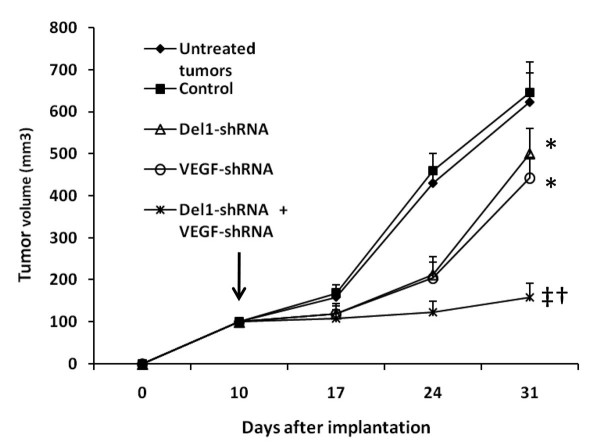
**shRNA gene therapy inhibits growth of tumors**. LS-174T tumors were established. When the tumors reached around 100 mm^3 ^(indicated by a vertical arrow), they were injected with control vector, Del1-shRNA, VEGF-shRNA, or a combination of Del1-shRNA plus VEGF-shRNA, and their sizes (mm^3^) were monitored and recorded. The untreated tumors also served as controls. A significant difference in tumor volumes from control is denoted by "*", and a highly significant difference by "†". "‡" Indicates significant difference from Del1-shRNA or VEGF-shRNA monotherapies.

Established LS-174T tumors (100 mm^3^) were intratumorally injected with either control vector, Del1-shRNA, VEGF-shRNA or Del1-shRNA + VEGF-shRNA expression plasmids complexed with FuGENE™ 6. The untreated tumors also served as control. The untreated tumors and the tumors treated with control vector grew remarkably fast reaching 623.1 ± 68.9 mm^3 ^and 645.2 ± 74.5 mm^3 ^in volume, respectively, three weeks after treatments. In contrast, tumors treated with Del1-shRNA or VEGF-shRNA plasmids reached only 501.7 ± 60.3 or 443.6 ± 47.8 mm^3 ^in volume, respectively, at the same time point (Figure 3). A combination of Del1-shRNA and VEGF-shRNA further suppressed tumor growth such that tumors reached only 158.4 ± 33.9 mm^3 ^in volume (Figure 3), indicating the synergistic effects of Del1-shRNA and VEGF-shRNA.

### Del1-shRNA and VEGF-shRNA synergize to inhibit tumor angiogenesis

**Figure 4:**
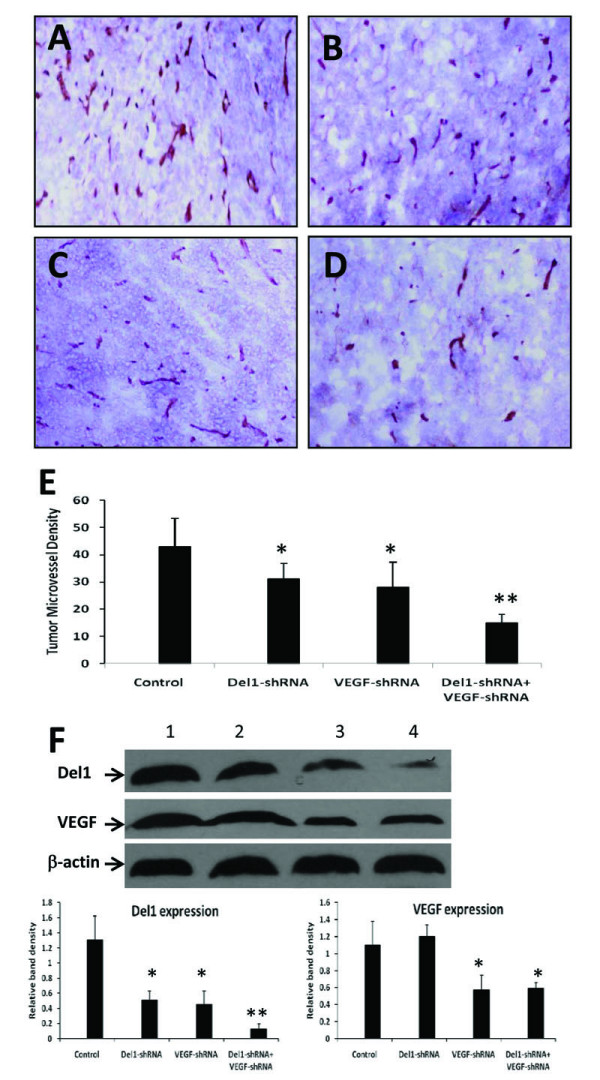
**shRNA gene therapy inhibits tumor angiogenesis by downregulating angiogenic factors**. (A-D) Illustrated are representative tumor sections prepared 3 weeks following intratumoral injection of either control vector (A), Del1-shRNA (B), VEGF-shRNA (C), or a combination of Del1-shRNA + VEGF-shRNA plasmids (D). (E) Tumor microvessels were stained with an anti-CD31 Ab and counted in blindly chosen random fields to record tumor microvessel density. A significant difference in microvessel counts in tumors treated with Del1-shRNA or VEGF-shRNA compared with control is denoted by "*", and a highly significant difference between Del1-shRNA + VEGF-shRNA versus control by "**". (F) Western blot analysis of Del1 and VEGF expression in tumors 4 days after injection with control vector (lane 1), Del1-shRNA (lane 2), VEGF-shRNA (lane 3), or Del1-shRNA + VEGF-shRNA (lane 4). Tumor homogenates were stained with Abs against Del1 (upper row), VEGF (middle row), and with an anti-β-actin Ab (lower row). The density of each band was measured and normalized to that of β-actin. *Indicates a significant difference at *P *< 0.05 in the band intensities of Del1 or VEGF between control vector transfected groups, and ** indicates significant difference from Del1-shRNA or VEGF-shRNA treated tumors.

LS-174T tumors established in mice were removed 3 weeks after intratumoral injection of control vector, Del1-shRNA, VEGF-shRNA or Del1-shRNA + VEGF-shRNA expression plasmids, and sectioned. The sections were stained with an anti-CD31 Ab revealing that both Del1-shRNA (Figure 4B), and VEGF-shRNA (Figure 4C) reduced tumor microvessel density by 28% and 35% (Figure 4E), respectively (both *P *< 0.05), compared with control (Figure 4A). The combinational therapy with Del1-shRNA + VEGF-shRNA (Figure 4D) was the most effective of all, as it reduced tumor microvessel density by 65% (*P *< 0.01), compared with control (Figure 4E), indicating that Del1-shRNA and VEGF-shRNA therapies have a synergistic effect in inhibiting tumor angiogenesis. Therefore, we detected the expression of both Del1 and VEGF by Western blot analysis, which revealed that gene transfection of Del1-shRNA had no effect on VEGF expression but VEGF-shRNA therapy downregulated expression of Del1, and synergized with Del1-shRNA to almost completely block the expression of Del1 (Figure 4F).

### Del1-shRNA and VEGF-shRNA synergizes to inhibit tumor cell proliferation *in situ*

**Figure 5:**
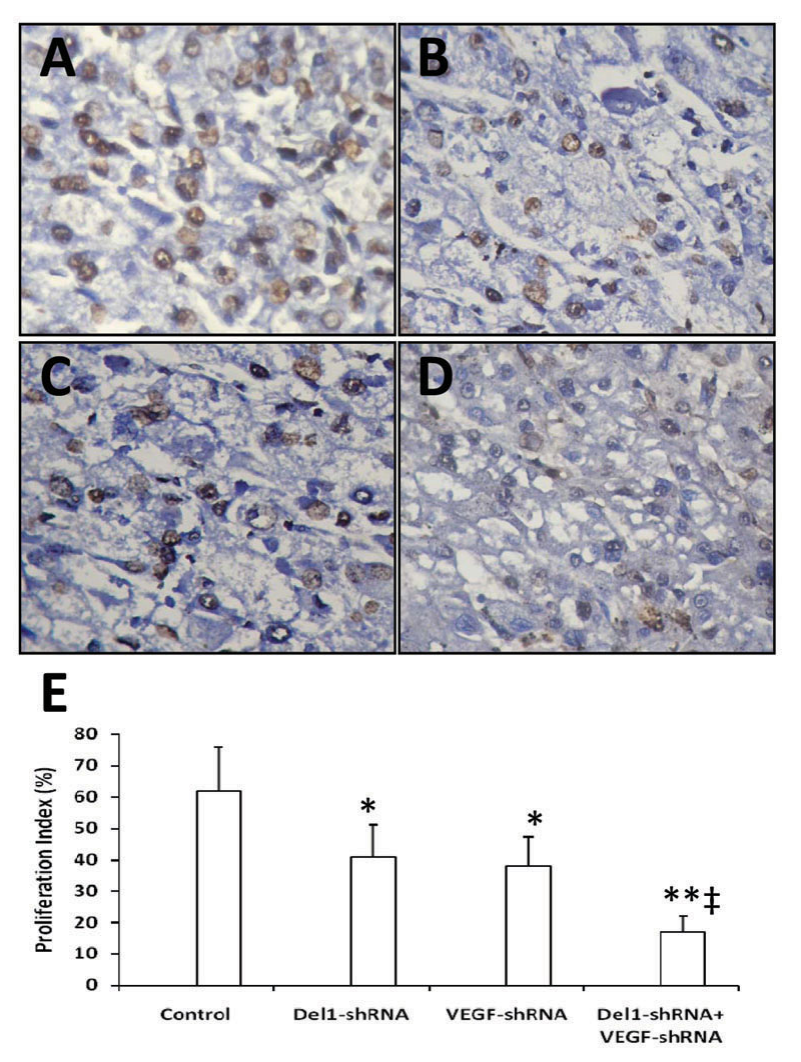
**shRNA gene therapy inhibits tumor cell proliferation *in situ***. Illustrated are representative tumor sections prepared 3 weeks after treatment from mice receiving either control vector (A), Del1-shRNA (B), VEGF-shRNA (C), or a combination of Del1-shRNA plus VEGF-shRNA plasmids (D). Tumor sections were stained with anti-Ki-67 Ab to detect proliferative cells. (E) The Ki-67 positive cells were counted to calculate the proliferation index. A significant difference in proliferation index between tumors treated with Del1-shRNA or VEGF-shRNA compared with control is denoted by "*", and a highly significant difference between Del1-shRNA + VEGF-shRNA versus control by "**", and a significant difference between the combinational therapy and Del1-shRNA or VEGF-shRNA monotherapies by "‡".

We next investigated whether shRNA gene therapy could inhibit cell proliferation in LS-174T tumors *in situ*. The tumor sections from above were stained with an anti-Ki-67 Ab. There were fewer Ki-67 positive cells in tumors treated with Del1-shRNA (Figure 5B) or VEGF-shRNA (Figure 5C) plasmids, compared with control vector-treated tumors (Figure 5A); and there were even fewer Ki-67 positive cells in tumors treated by the combinational therapy with Del1-shRNA + VEGF-shRNA plasmids (Figure 5D), compared with tumors treated by the monotherapies. Ki-67 positive cells in sections were counted to record proliferation index. Gene transfer of Del1-shRNA resulted in a significant 31% (*P *< 0.05) reduction in proliferation index compared with control, and VEGF-shRNA therapy also reduced proliferation index by 42% compared with control (*P *< 0.01). Furthermore, the combinational therapy with Del1-shRNA + VEGF-shRNA plasmids highly significantly reduced the proliferation index by 71%, compared with mock treatment (P < 0.001) (Figure 5E).

### Downregulation of Del1 by Del1-shRNA does not alter viability of colon cancer cells *in vitro*

**Figure 6:**
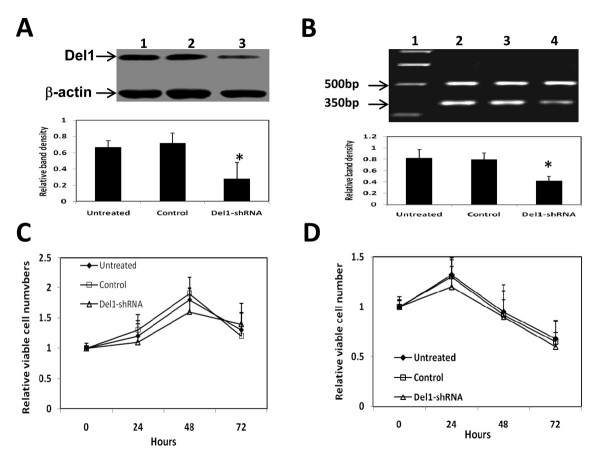
**Del1-shRNA downregulates Del1 expression but has no effects on cell viability *in vitro***. (A) Del1 protein (upper row) in lysates of untreated LS-174T cells (lane 1) or cells transfected with control vector (lane 2), or Del1-shRNA plasmid (lane 3) was Western blotted. The density of each band was measured and compared to that of the internal control β-actin (lower row). (B) Del1 mRNA (350 bp) expression detected by RT-PCR in lysates of untreated LS-174T cells (lane 2) or cells transfected with control vector (lane 3), or Del1-shRNA plasmid (lane 4). G3PDH (500 bp) was amplified as a PCR internal control. Lane 1 denotes the DNA marker. * Indicates a significant difference at *P *< 0.05 in the band intensities of Del1 compared to control. (C) The LS-174T cells were further cultured in medium supplemented with %FCS, or (D) without FCS, and harvested at indicated time points. The viability of cells was assessed by the MTT method.

The LS-174T cells were transfected with Del1-shRNA expression plasmids, harvested 48 h later, lysed and subjected to Western blot analysis. As shown in Figure 6A, Del-1shRNA gene transfection greatly downregulated expression of Del1 protein, which was supported by the downregulation of Del1 mRNA by RT-PCR (Figure 6B). A potential mechanism for the Del1-associated increase in tumor growth rate would be a Del1-mediated growth or survival advantage conferred directly on the cells [8]. Thus, we examined the viability of the Del1-shRNA-transfected cells. The transfected cells above were further cultured and harvested 24, 48 and 72 h later and assessed by MTT assay, which revealed that Del1-shRNA transfection had no effect on cell viability (Figure 6C). By using a simple viability assay, we further investigated downregulation of Del1 could affect tumor cell survival by depriving of serum, which makes cells susceptible to apoptosis. As shown in Figure 6D, there was no significant difference in the numbers of viable cells in untreated, control vector-transfected or Del1-shRNA-transfected cells after serum removal. We have also used the same methods above in another human colon cancer HT29 cells, and similar results were obtained (data not shown). Taken together, these data suggest that downregulation of Del1 by Del1-shRNA does not alter the mitotic index or the apoptotic index of the transfected tumor cells, supported by one previous report [7].

### The effects of VEGF-shRNA on Del1 expression *in vitro*

**Figure 7:**
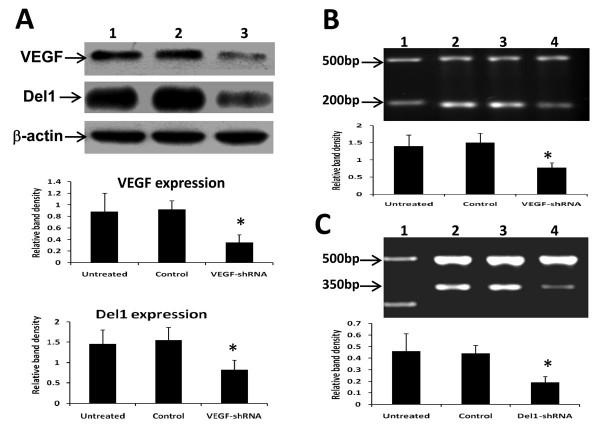
**VEGF-shRNA downregulates expression of VEGF and Del1 *in vitro***. LS-174T cells were transfected with VEGF-shRNA and harvested 48 h later. (A) VEGF (upper row) and Del1 proteins (middle row) in lysates of untreated cells (lane 1) or cells transfected with control vector (lane 2), or VEGF-shRNA plasmid (lane 3) was Western blotted. The density of each band was measured and compared to that of the internal control β-actin (bottom row). (B, C) The expression of VEGF mRNA (200 bp) (B) or Del1 mRNA (350 bp) (C) was detected by RT-PCR in lysates of untreated LS-174T cells (lane 2) or cells transfected with control vector (lane 3), or Del1-shRNA plasmid (lane 4). GAPDH (500 bp) was amplified as a PCR internal control. Lane 1 denotes the DNA marker. * Indicates a significant difference at *P *< 0.05 in the band intensities compared to control.

Give that VEGF-shRNA gene therapy downregulated expression of VEGF and Del1 *in situ*, we further investigated whether VEGF-shRNA has the same effects *in vitro*. The LS-174T cells were transfected with VEGF-shRNA expression plasmids and harvested 48 h later, lysed and subjected to Western blot analysis with Abs against VEGF and Del1, respectively. As shown in Figure 7A, VEGF-shRNA gene transfection significantly downregulated expression of both VEGF and Del1 proteins, which was further supported by the downregulation of VEGF mRNA (Figure 7B) and Del1 mRNA by RT-PCR (Figure 7C).

## Discussion

The present study has demonstrated that downregulation of Del1 with small interfering RNA gene therapy targeting Del1 and VEGF suppresses the growth of colon tumors by inhibiting angiogenesis and cell proliferation *in vivo*. Del1 was first identified on the basis of the early, transient, endothelial cell-restricted pattern of expression in embryo, suggesting that Del1 might have a role in the initiation of blood vessel formation. Del1 binds to integrin α_v_β_3_, an endothelial cell vitronectin receptor, mediating endothelial cell attachment and migration, as well as activation of the MAP kinase signaling cascade [12]. It is known that integrin α_v_β_3 _is essential for vascular development in tumor- and cytokine-induced angiogenesis as well as embryogenesis [13-16], and inhibition of integrin α_v_β_3 _has been shown to reduce tumor growth and metastasis through the disruption of tumor angiogenesis [10,17-19]. While α_v_β_3 _ligands such as vitronectin are clearly important for supporting angiogenesis [5,16], it has also been observed that Del1 supports proliferation of endothelial cells through inhibition of apoptosis [12]. Overexpression of Del1 has been shown to accelerate tumor growth by enhancing vascular formation [7]. These data indicate that Del1 could be a potential target for anti-angiogenic therapy. However, the role of Del1 in tumor development and progression has not been so well studied as its receptor, integrin α_v_β_3_. It has been demonstrated that Del1 is expressed in some forms of cancer tissues [7] and tumor cell lines [6,8]. Aoki reported that Dunn osteosarcoma and murine sarcoma cells did not express Del1 *in vitro*, but the expression of Del1 was observed after subcutaneous inoculation *in vivo *[9]. The present study shows here that the human colon cancer LS-174T and HT29 cells expressed Del1. The difference in Del1 expression may be cell type-dependent, as previously reported that the Del1 expression was obviously diverse in various tumor cells [6,8].

In the present study, we used shRNA expression vector to downregulate expression of Del1 and VEGF, as it allows long lasting and more stable gene silencing after gene transfection [20]. In the Del1-shRNA expression vector, four pieces of siRNA targeting four sites of Del1 gene were introduced, and in VEGF-shRNA vector, three siRNAs were inserted. Gene transfer of Del1-shRNA reduced the expression of Del1 protein by 60% *in vivo*, and VEGF-shRNA, 55% of VEGF protein levels, compared with controls. The downregulation of the two angiogenic factors exhibited anti-tumor activity and displayed a synergistic effect in suppressing growth of established colon tumors by inhibiting tumor angiogenesis and cell proliferation. However, Del1-shRNA did not show significant effect on the viability of the cultured tumor cells though it similarly downregulated expression of Del1 protein and mRNA of cells *in vitro*. The results may suggest that the therapeutic effect of Del1-shRNA was provided by the decreased tumor vascularization resulting from Del1-mediated endothelial cell attachment, migration, and support of proliferation, rather than direct anti-proliferative effect on tumor cells. The inhibition of neovascularization by Del1-shRNA may restrict the supply of tumor cell survival factors provided either by endothelial cells or by the circulation. The mechanism by which Del1-shRNA inhibits tumor cell proliferation may be due, in part, to the loss of an adequate vasculature, which would deprive the tumor of oxygen and nutrients. Some endothelial-cell-derived paracrine factors have been reported to promote cell survival, including Platelet-derived growth factor, IL-6 and heparin-binding epithelial growth factor [21]. Production of paracrine factors is decreased, in part, because angiogenesis inhibitors can inhibit endothelial-cell proliferation [22].

VEGF, the key mediator of angiogenesis, is a potent endothelial mitogen and permeability factor, commonly expressed in tumors [23]. VEGF strongly induces the activity of extracellular signal-regulated kinases 1 and 2, which play a central role in the stimulation of endothelial cell proliferation [24]. VEGF has been implicated as a critical molecular signal in tumor development, by promoting angiogenesis [25], suppressing anti-tumor immune response [23,26], and possibly exerting autocrine functions on tumor cells [27,28]. It has been reported that VEGF contributes to mammary tumor growth through paracrine and autocrine mechanisms [29,30]. Therefore, by downregulating VEGF, VEGF-shRNA could directly inhibit proliferation of tumor cells. To date, the relationship between VEGF and Del1 remains unclear. A number of factors secreted from tumor cells, such as IL-1, tumor necrosis factor-α and VEGF, induce the expression of Del1 in vascular endothelial cells [9]. But the exact molecular pathway accounting for the interaction between VEGF and Del1 in tumor cells needs further investigation, which might improve our understanding of the role of Del1 in tumor angiogenesis, and optimize Del1-targeted cancer therapy.

## Competing interests

The authors declare that they have no competing interests.

## Authors' contributions

XZ carried out construction of shRNA expression vectors, immunohistochemical analysis, and *in vitro *assays, participated in animal experiments and drafted the manuscript. HQ carried out the animal experiments and participated in designing the study and coordination. XJ carried out RT-PCR, and participated in immunohistochemical analysis and animal experiments. XD carried out Western blot analysis. HJ participated in the design of the study and performed the statistical analysis. XS conceived of and designed the study, participated in its coordination, and finalized the manuscript. All authors read and approved the final manuscript.
